# Heart Rate Variability Responses to an Undulating Resistance Training Program in Free-Living Conditions: A Case Study in a Collegiate Athlete

**DOI:** 10.3390/sports6040121

**Published:** 2018-10-20

**Authors:** Clifton J. Holmes, Stefanie A. Wind, Michael R. Esco

**Affiliations:** Department of Kinesiology, The University of Alabama, Tuscaloosa, AL 35487, USA; stefanie.wind@ua.edu (S.A.W.); mesco@ua.edu (M.R.E.)

**Keywords:** heart rate variability, coefficient of variation, strength training

## Abstract

The purpose of this case study was to evaluate the response in heart rate variability via the parasympathetically-mediated metric of the log-transformed root mean square of successive R-R interval differences (lnRMSSD) to weekly variations in total volume-load (TVL) during an 18-week periodized strength training program in a competitive collegiate hockey athlete. The program consisted of three 60–90 min full-body exercise sessions per week with at least 24-h of rest between each session. Daily lnRMSSD measurements were taken immediately after waking using a validated smartphone application and the pulse-wave finger sensor. The weekly lnRMSSD values were calculated as the mean (lnRMSSD_MEAN_) and the coefficient of variation (lnRMSSD_CV_). A Pearson’s bivariate correlation of lnRMSSD_MEAN_ and TVL revealed no statistically significant correlation between the two variables; TVL (*r* = −0.105, *p* = 0.678). However, significant correlations were found between lnRMSSD_CV_ and both total load (TL) (*r* = −0.591, *p* = 0.013) and total volume (TV) (*r* = 0.765, *p* < 0.001). Additionally, weekly ratings of perceived exertion (RPE) mean values were statistically significantly correlated to TVL, *r* = 0.853, *p* < 0.001. It was concluded that lnRMSSD_CV_ increased or decreased proportionally to an increase or decrease in TVL during the periodized resistance training program with TV being the strongest, independent indicator of these changes.

## 1. Introduction

Heart rate variability (HRV) is defined as the oscillations that occur between successive heart beats and is considered a noninvasive marker of autonomic nervous control of the cardiovascular system [1]. Though it has been traditionally measured within clinical settings as a prognostic indicator of cardiovascular-related diseased states [2], recent research has supported the use of HRV as an objective, physiological indicator of stress and recovery to variations in training load among athletes [3,4,5]. However, the standard requirements of electrocardiogram and extended recording periods of at least 10 min for short-term assessment have made it difficult to measure HRV in field settings. Fortunately, several HRV smartphone devices have been validated in the literature for acquiring the parasympathetically-derived marker of the root mean square of successive R-R intervals (RMSSD) in free-living conditions [6,7]. This has been considered the preferred HRV marker for athletic monitoring primarily because it can be accurately measured with an ultra-shortened recording of only 1-min [8] following a 1-min stabilization period [9]. The emerging smartphone technology has allowed convenience of following recent recommendations of acquiring daily RMSSD throughout a week then averaging (i.e., RMSSD_MEAN_) to derive more meaningful information about training status compared to isolated recordings [6,10,11]. Furthermore, smartphone applications are useful for quantifying daily fluctuation of RMSSD as assessed by the coefficient of variation (RMSSD_CV_) which represents perturbations to cardiac-autonomic homeostasis [6]. 

When attempting to track long-term training responses and adaptations, weekly HRV changes specifically in RMSSD_MEAN_ and RMSSD_CV_, have been shown to be useful indicators. A decreased RMSSD_MEAN_ and an increased RMSSD_CV_ is correlated with lower aerobic fitness [12,13], higher perceived fatigue and increased training load in team sports [9,13]. Additionally, higher RMSSD_MEAN_ was related to a smaller RMSSD_CV_ (*r* = −0.53), which suggests that greater parasympathetic activity reflects a higher resilience or capacity to training stress [14]. With the growing body of literature around RMSSD trends seen in athletes, coaches, exercise physiologists, and various other practitioners can use HRV data to evaluate individual responses and potentially guide future training.

Though HRV is a promising, objective method for tracking responses in athletes, most of the research has focused on aerobic training with few studies examining the effects of long-term resistance training. In addition, the majority of the research done thus far has focused on mean data from an entire group. The limitation of such research designs is that individual responses are often overlooked. Individual differences are a significant factor when measuring fatigue accumulation and tracking recovery. A case study format allows for a more detailed description of physiological outcomes to training among individual athletes, which is especially beneficial in regard to HRV alterations following a periodized resistance training program since the relationship between HRV following resistance training is not well understood. Case studies have been utilized previously to provide a more in-depth examination of the exercise prescription and performance factors that influence HRV in endurance trained athletes [15,16]. Therefore, the purpose of this case study was to evaluate the response in HRV (i.e., RMSSD_MEAN_ and RMSSD_CV_) to weekly variations in total volume-load (TVL) during an off-season 18-week periodized strength training program in a collegiate athlete. Additionally, this case study looked to examine which component of TVL, total load (TL), or total volume (TV), had a more significant effect on HRV responses. The hypothesis was, first, that RMSSD_MEAN_ would decrease and RMSSD_CV_ would increase with greater TVL. 

## 2. Methods

This case study was conducted in a collegiate male athlete during the off-season in a free-living condition. The athlete participated in an 18-week periodized strength training program. Throughout the program, weekly adjustments in training load were made. The program consisted of three 60–90 min full-body exercise sessions per week with at least 24-h of rest between each session. Though the athlete was provided a training program to adhere to, no other parameters or lifestyle factors were controlled for that may have affected HRV. For these reasons, the current case report is considered a “free-living” scenario in comparison to traditional HRV research studies done in laboratory settings. It is well understood that HRV can be affected by any number of external stressors experienced by an individual, specifically additional physical activity and sleep deprivation. Unfortunately, it was beyond the scope of this study to control for these variables as the purpose was to specifically allow for the collection of HRV measures in a free-living condition. Daily RMSSD measures were taken from an ultra-shortened recording using a validated smartphone application and the pulse-wave finger sensor (PWFS) throughout the training program. For each week, RMSSD_MEAN_ and RMSSD_CV_ were determined. Measures were taken immediately upon waking each morning. The athlete was instructed to use the restroom prior to the recording and to avoid the ingestion of any food or drink beforehand. All measurements were done in a seated position after at least a 1-min stabilization period. During every training session, TVL was recorded as the combination of TV and TL. These studied variables were recorded during each week of the training program. 

One athlete (ht = 183.9 cm, wt = 86.8 kg, age = 22.7 years), who is a member of the University’s club hockey team, volunteered for this case study. Prior to participating in the study, the athlete provided written informed consent that was approved by the Institutional Review Board for Research in Human Participants. The athlete was given a Physical Activity Readiness Questionnaire (PAR-Q) and an additional short questionnaire to assess health history, readiness to train, and training background. The athlete was classified “advanced” based on the criteria of the National Strength and Conditioning Association (NSCA) [17], since he had eight years of consistent resistance training at least three days a week. 

The resistance training program was designed and supervised by the main author who is a NSCA Certified Strength and Conditioning Specialist. The 18-week program was split into four phases labeled as “mesocycles”. Before beginning the first mesocycle, the athlete completed a familiarization session where he was instructed on proper techniques for all the exercises to be performed throughout the program. After familiarization was completed, eight repetition-maximum (RM) testing was done for the primary exercises of the program: back squat, bench press, and conventional deadlift. Testing was performed during three separate sessions over the course of one week with at least 24 h of rest between each session. The results of the initial pretesting 8RM testing were used to estimate 1RM for each exercise and then assign loads for the following training weeks based on percentages of 1RM. At the end of the second mesocycle, which was termed as the “mid-point” of the program, the athlete completed another 8RM test for each exercise in order to track and adjust for any strength adaptations. The results of the 8RM testing were again used to estimate 1RM for each exercise and then assign loads for the following training weeks based on percentages of 1RM. At the end of the 18-week program, a post-test 3RM for each exercise was performed and 1RM was again estimated to analyze changes in strength in comparison to the previous measures. All 1RM estimations were based on the NSCA “Estimated 1RM and Training Loads” table [17] derived from the Lander’s formula [18]: (100 × Weight) ÷ (101.3 − (2.67123 × Number of repetitions)). The results of 1RM estimation testing at each point can be found in Table 1. The 1RM was not used in order to avoid the increased risk of injury which could have negatively impacted the athlete’s ability to perform in the upcoming season. Strong correlations have been shown between 1RM and *n*RM [19,20,21,22]. It was also determined that two different repetition-maximum tests (8RM and 3RM) should be used in order to better reflect muscular adaptations made during the different training mesocycles (i.e., high-repetition, low-intensity vs. low-repetition, high-intensity) [23]. 

The first two mesocycles were five weeks each consisting of a 4:1 paradigm: four weeks of progressive overload increasing both load and volume. The fifth week was a “deload” week where volume and weight are reduced in order to allow the athlete to recover from accumulated fatigue. The third mesocycle consisted of a 3:1 paradigm with three weeks of progressive overload followed by a fourth week of deload. The fourth and final mesocycle consisted of four continuous weeks of progressive overload through the increase in load primarily. At the completion of this mesocycle, the athlete was allowed a one week rest period of no training. Following the recovery week, 3RM testing of each primary exercise was performed. 

The first two mesocycles were designed to have high training volumes and low-to-moderate training loads, while the second two mesocycles were designed to have moderate-to-low training volumes and moderate-to-heavy training loads. For the first two mesocycles, three and six sets of eight to 12 repetitions were performed per exercise with training load percentages varying between 50 to 77% of 1RM. For the second two mesocycles, three to six sets of three to six repetitions were performed per exercise with training load percentages varying between 70 to 90% of 1RM. Ratings of Perceived Exertion (RPE) using the Borg CR-10 scale was collected after each training session as a means of measuring intensity and a subjective measure of fatigue accumulation. RPE was used in addition to 1RM percentage loading zones to determine intensities of the prescribed training sessions, as well as the athlete’s perception of performance [24,25,26,27]. Intra-set rest periods were 60–90 s for the first mesocycle, two minutes for the second mesocycle, two to three minutes for the third mesocycle, and three minutes for the fourth mesocycle. Each training session was designed to be a full-body workout with exercises consisting of the following general approach: a primary exercise, a posterior chain/hip dominate movement, a push, a pull, a single-leg exercise, and a core strengthening movement. A more specific list of the exercises used throughout the program separated by mesocycle and session can be found in Table 2. 

All data concerning exercises, rest periods, volume, load, and total volume-load were written down by the coach during each individual session. Total volume-load was calculated as the product of the number of total repetitions of all exercises (i.e., TV) multiplied by the total weight lifted for all exercises (i.e., TL). The number of repetitions, number of sets, and the load used were recorded for each exercise during each session. Volume was calculated for each exercise (sets multiplied by reps) during a single session. Then the load used for that exercise was multiplied by the volume in order to get the volume-load for that exercise. This process was repeated for each exercise within a session and then the total volume-load for the entire session was summed up. Each volume-load per session within a week were summed together to get the total volume-load for that week. Total volume was calculated as the sum of all repetitions done for all exercises and total load was calculated as the sum of all weight lifted for all exercises within each session for one week. 

The HRV data collection procedures were based on the methods researchers have reported in previous studies involving a group of collegiate athletes using similar training programs [28]. HRV data was self-measured daily by the athlete with a validated smartphone application and optical pulse-wave finger sensor (PWFS) apparatus (i.e., photoplethysmograph) that inserts in to the headphone slot of a mobile device (HRV Fit Ltd., Southampton, UK). This device has been shown to provide accurate RMSSD analysis compared with electrocardiography [29,30]. In addition, the PWFS measures RMSSD following an ultra-short time period of 1-min stabilization period followed by a 1-min recording period, which has been shown to provide valid measures compared to the standard 5-min stabilization and 5-min recording period [8,31]. Each morning after waking and elimination, the subject performed a seated HRV recording by inserting his left index finger into the PWFS. The subject initiated the RMSSD recording while remaining motionless, breathing spontaneously, and with their left hand held still, within 20 cm from the left side of their chest. According to the ithlete^TM^ manufacturer (HRV Fit Ltd. Southampton, UK), the application modifies the RMSSD by taking the natural log transformation and multiplying by twenty providing a more intuitive and interpretable figure for the user on a ~100 point scale. RMSSD_CV_ is calculated from the weekly RMSSD standard deviations divided by weekly RMSSD mean then multiplied by 100 to produce a percentage [5].

All statistical procedures were performed with SPSS 23.0 (IBM Corp., Armonk, NY, USA). Weekly data (HRV and TVL) were expressed as means ± standard deviations and sum totals. Pearson’s bivariate correlations were done to determine the relationships between the dependent (RMSSD_MEAN_ and RMSSD_CV_) and independent (TVL, TV, and TL) variables. For further examination following a statistically significant correlation being found between the variables, multiple regression analysis using forward variable selection methods were conducted to determine which training variable (i.e., TL versus TV) was independently associated above and beyond the other with RMSSD_MEAN_ and RMSSD_CV_. Significance for correlations and regression procedures was set at *p* < 0.05.

## 3. Results

**Figure 1:**
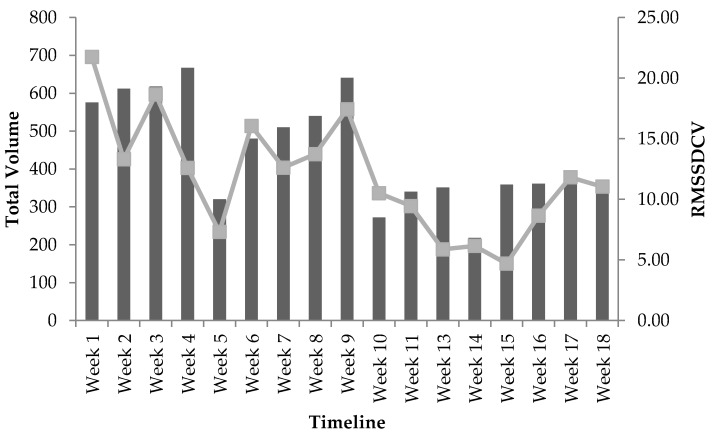
The responses in RMSSD_CV_ in relation to total volume across the 18-week training period: bars represent total volume; line represents RMSSD_CV_ values; and mesocycle 1 (W1–W5), mesocycle 2 (W6–W10), mesocycle 3 (W11–W14), and mesocycle 4 (W15–W18).

**Figure 2:**
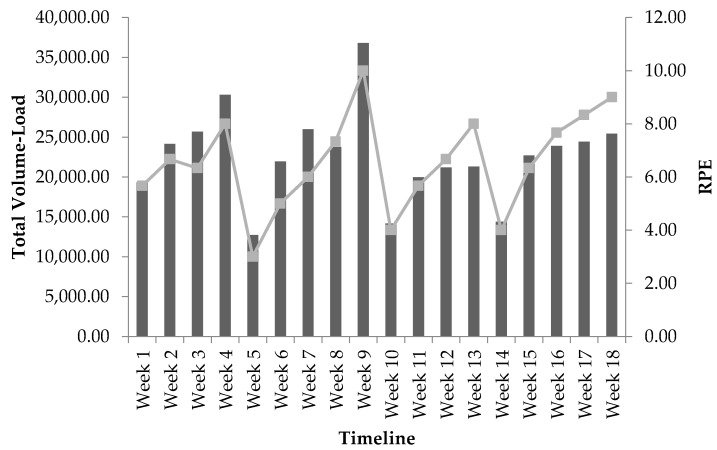
The responses in RPE in relation to total volume-load across the 18-week training period: bars represent total volume-load; line represents RPE values; and mesocycle 1 (W1–W5), mesocycle 2 (W6–W10), mesocycle 3 (W11–W14), and mesocycle 4 (W15–W18).

Only 17 weeks of RMSSD data was obtained to calculate because of technical issues with the equipment at week 12. Progression of weekly measures recorded for TVL and RMSSD at baseline and throughout the 18-week program can be seen in Table 3. Progression of weekly mean RPE values recorded throughout the 18-week program can be seen in Table 4. According to the bivariate correlation procedures, RMSSD_MEAN_ was not statistically significantly related to TVL (*r* = −0.105, *p* = 0.678), TL (*r* = −0.343, *p* = 0.164), or TV (*r* = 0.048, *p* = 0.851) throughout the 18-week program. RMSSD_CV_ was not statistically significantly related to TVL (*r* = 0.421, *p* = 0.093), however, significant correlations were found with both TL (*r* = −0.591, *p* = 0.013) and TV (*r* = 0.765, *p* < 0.001). Following this finding, a multiple regression analysis using the forward method order indicated that TV was a stronger predictor of RMSSD_CV_ (*r* = 0.765, *p* < 0.001) than for TL, accounting for approximately 59% (*r^2^* = 0.585) of the variance; a partial correlation of TL and RMSSD_CV_ was not found to be significant, *r* = −0.187, *p* = 0.488. The responses in RMSSD_CV_ with the adjustment in TV across the training program are shown in Figure 1. Additionally, weekly RPE was statistically significantly correlated to TVL, *r* = 0.853, *p* < 0.001. The responses in RPE with the adjustment in TVL across the training program are shown in Figure 2.

## 4. Discussion

The purpose of this case study was to examine the relationship between training indicators (TVL, TL, and TV) and weekly RMSSD values (i.e., mean and CV) collected within a free-living condition in a collegiate athlete during an 18-week resistance training program. The results demonstrated that the change in RMSSD_MEAN_ throughout the program was not statistically related to the changes in TVL, TL, or TV. However, RMSSD_CV_ response was statistically significantly correlated to the variation in TV and TL. This significant finding was followed by a multiple regression analysis using the forward method to more precisely determine which training indicator of TVL (i.e., TL vs. TV) was independently related to RMSSD_CV_. The results indicated that TV was the only variable independently associated to RMSSD_CV_. The strong correlation (*r* = 0.765) and the *r^2^* value of 0.585 indicated that the variation in TV explained approximately 59% of the variation in RMSSD_CV_ that occurred throughout the 18-week program. However, TL was not independently associated, above and beyond that of TV, to RMSSD_CV_. 

Previous research has reported no significant changes in HRV following long-term resistance training [32,33,34,35]. For instance, Cooke and Carter suggested that an 8-week resistance training program does not affect cardiac-vagal HRV metrics in young healthy subjects [32]. However, the pre-versus post-HRV responses to the resistance training programs in the previous research were measured under isolated recording conditions of only one day at each time point [32,33,34,35,36]. In the current study, RMSSD was measured daily, with the exception of week 12 because of the technical difficulties with the athlete’s smartphone. The weekly mean values (e.g., RMSSD_MEAN_) fluctuated on a week-to-week basis. For instance, when compared to baseline, there was a less than 1% change in RMSSD_MEAN_ at weeks 1 and 10, but a greater than 10% change in RMSSD_MEAN_ at weeks 8 and 17 (complete week-to-week analysis is shown in Table 3). Wide spread fluctuations of RMSSD_MEAN_ demonstrate the flaws of isolated pre-versus post-measurements in longitudinal HRV studies. These changes are representative of shifts in overall cardiac-parasympathetic control throughout the training program. The week-to-week responses in RMSSD_MEAN_ may be mostly related to longitudinal changes in aerobic fitness rather than improvements in muscular performance by long-term resistance training [32,33,34,35,36]. For instance, Esco et al. showed that the initial change in RMSSD_MEAN_ from weeks 1 to 3 of an 11-week conditioning program was strongly related to the adaptation of maximal oxygen consumption in female soccer player [37]. Though maximal oxygen consumption was not accounted for in the case study, future research should analyze the impact of aerobic fitness on muscle performance recovery and HRV responses in resistance trained athletes.

Most noteworthy, RMSSD_CV_ showed large fluctuations from week-to-week throughout the training program and the dramatic change from one mesocycle to the next. These changes appeared to reflect changes in TV, since it provided the strongest independent relationship to RMSSD_CV_. The 18-week resistance training program was divided into four mesocycles that generally followed an undulating model as follows, high TV with low-to-moderate TL with the first two mesocycles (i.e., weeks 1 to 10) and high TL with low-to-moderate TV with the second two mesocycles (weeks 11 to 18). Indeed, the highest lnRMSSD_CV_ measures were seen in the first two mesocycles that contained the highest TV and decreased during the last two mesocycles with the lowest TV. From a week-to-week perspective, the decreases in RMSSD_CV_ were shown during or following weeks of substantial reduction in TV (e.g., weeks 5, 10, and 14). For example, at week 1, RMSSD_CV_ was 33% greater than baseline reflecting how unaccustomed the athlete was to the programing. Conversely, by week 4, RMSSD_CV_ had dropped 23% from baseline showing the adaptation and familiarity with the training sessions. Furthermore, at week 11, the first week of the low TV and moderate to high TL mesocycles, RMSSD_CV_ was 42% lower than baseline. These findings are supported by a previous study that showed large to very large increases in RMSSD_CV_ during two weeks of overload training followed by decreases in the 2-week tapering period in competitive sprint swimmers [28]. The overload and tapering was performed by increasing or decreasing the volume of training that occurred in the water [28]. Therefore, the week-to-week change in RMSSD_CV_ was likely due to a homeostatic response related to fatigue generated by changes in TV during each week of resistance training over the course of the program [38], and supports RMSSD_CV_ for serving as an objective indicator of recovery status.

Case studies are often utilized to provide an in-depth examination into responses to training in individual athletes. Designs such as these have provided useful information regarding HRV changes in specific athletes. For instance, a near perfect correlation (*r* = −0.92) was shown between RMSSD_CV_ and 8-km performance in a cross-country athlete of another case study [15]. This suggests that increases and decreases in RMSSD_CV_ in the weeks preceding competition may reflect poorer and better performance, respectively, in individual athletes [15]. Plews et al. reported a case comparison between two elite triathletes over a 77-day period leading to competition. One athlete performed poorly in the triathlon due to being diagnosed with nonfunctional overreaching and subsequently acquired the shingles virus [16]. In this athlete, large decreases in both RMSSD_MEAN_ and RMSSD_CV_ were shown leading to competition, while these variables remained stable in the other athlete who performed well in competition and remained healthy [16]. These findings suggest that trends in both mean and CV of HRV may be useful for indicating positive or negative adaptation with training. 

It is well-known that as HR increases, HRV decreases, but sympathetic activity does not completely engage until approximately 100 bpm or more [39,40]. Without repeatedly reaching this point multiple times in training, it is unlikely that any adaptations will occur in autonomic modulation. Cardiac output (the product of heart rate and stroke volume) responses to more intense resistance training have been reported by Lentini and colleagues, who had healthy male subjects perform a double leg press to failure at 95% of their maximum dynamic strength [41]. Cardiac output increased significantly during the lifting phase and increased further during the lockout phase. The increase in cardiac output, however, is modest compared to that with aerobic exercise—and is due almost entirely to an increase in heart rate, which reached approximately 140 bpm, as stroke volume was relatively unchanged or decreased slightly during the exercise [39]. Many studies indicate that heart rate increases modestly during resistance exercise to volitional fatigue. When taken to volitional failure, low load resistance exercise results in a larger volume of work being done, producing heart rates more elevated than for a single 1RM [39,42,43]. Some authors, however, have reported greatly elevated heart rates during high-intensity resistance exercise [39,44]. Heart rate increases during acute resistance exercise are due to vagal withdrawal and stimulation of the sympathetic nervous system.

Autonomic responses to resistance training depend on the external stress created by the exercise being done. If there is not a sufficient amount of stress placed on the body and homeostasis is not greatly disturbed, then no significant autonomic modulation can occur. Melo et al. and Takahashi et al. both only used eccentric knee flexion and extension and saw no increases in HRV [45,46]. Heffernan et al. only conducted 6-weeks of training with undisclosed load which can be assumed to be light-to-moderate; they also had a 4-week detraining period after so all HRV improvements were lost [47]. Kanegusuku et al. used very low volume, long rest periods, and did not detail how overload was applied; no improvements in HRV were observed [34]. In the present study, total volume demonstrated stronger correlations with RMSSD_CV_ than total load. A potential explanation for this could be the greater increases in heart rate and longer durations of sympathetic activity during each set of each exercise performed during the training sessions. The higher repetition ranges of the first two mesocycles required more time under-tension allowing for greater elevation in heart rate in comparison to the smaller repetition ranges and lower overall volumes in the last two mesocycles (e.g., a set of 10 reps versus a set of 3 reps). Additionally, shorter rest periods were used in the first two mesocycles than in the final two mesocycles, which means that heart rate there would be less time for parasympathetic modulation to decrease heart rate. Multiple high-volume sessions week-to-week with these acute cardiovascular responses could have led to the observed fluctuations in RMSSD_CV_.

To increase muscular fitness and hypertrophy progressive overload must be applied. However, a periodized model should be followed in order for an athlete to continuously improve and prevent nonfunctional overreaching or the plateau effect. Therefore, any disruption in homeostasis must be performed in a methodical manner to meet the specific goals of a given training cycle. Most approaches follow a standard format for all athletes as objective methods to gauge individual responses are limited. However, in two separate studies Kiviniemi et al. demonstrated the effectiveness of HRV-guided, periodized aerobic training on performance outcomes in endurance trained athletes and previously sedentary subjects [48,49]. Subjects in the HRV-guided groups improved endurance performance significantly more than the subjects that followed the standard approaches [48,49]. The results of this case study suggest that HRV may also be useful for guiding periodization programs for strength and conditioning. Increases in RMSSD_CV_ should hypothetically indicate that the adaptive response has been initiated with an increase in training volume, such as at the beginning of a preparatory phase [17]. As training continues, the decrease in volume and subsequent increase in training load should be verified by a decrease in RMSSD_CV_. Throughout the training program, RMSSD_MEAN_ should remain consistent to confirm the prevention of nonfunctional overreaching or increase if an improvement in aerobic fitness is desired [16]. If such responses are not seen, then the strength and conditioning practitioner may need to consider an adjustment in load, volume, or other prescriptive factors until the desired week-to-week responses in HRV are demonstrated according to the perspective long-term goals of the specific training cycle. In the present study, RPE was shown to have significant correlation with total volume-load, but not with HRV metrics. RPE has been shown to be a strong indicator of fatigue accumulated during resistance training programs [25,26,27,28,29,30]. The combination of subjective measures from RPE and objective measures from HRV metrics may be a viable option for measuring fatigue accumulation and tracking recovery for researchers and practitioners. 

Though the findings of this case study are novel, further investigation is needed before any recommendations can be made. Caution should be employed when generalizing the findings toward resistance training among an entire group of athletes. As stated earlier in this paper, there is number of individual differences between athletes that may affect the amount of fatigue accumulated and the timeframe for proper recovery. Due to the individualized nature of HRV, a systematic investigation is needed to expand the foundation that was provided with the current case study and provide a more thorough understanding on how HRV responses should be interpreted to adjustments in resistance training stimuli among groups of athletes. 

Measurement of aerobic fitness was beyond the scope of this study. In order to get a more accurate depiction of the relationship between resistance training and HRV responses, the participant was not prescribed any aerobic training. Future studies should examine the effects of concurrent aerobic and resistance training. Another limitation of this study was the observational nature and free-living circumstance of data collection. Though the athlete was provided a training program to adhere to, no other parameters or lifestyle factors were controlled for that may have affected HRV. For these reason, the current case report is considered a “free-living” scenario in comparison to traditional HRV research studies done in laboratory settings. It is well understood that HRV can be affected by any number of external stressors experienced by an individual, specifically physical activity and sleep. Unfortunately, it was beyond the scope of this study to control for these variables as the purpose was to specifically allow for the collection of HRV measures in a free-living condition. That being said, in order to fully take HRV measures from lab settings to field settings, more free-living and mobile scenarios must be investigated with daily measures being taken without controlling for cofounders often performed in laboratory settings. Future research should look to track these variables while still maintaining free-living settings. 

## 5. Conclusions

HRV is an emerging training status metric being used for objectively monitoring athletes across a training period. However, most of the previous research has occurred relative to aerobic exercise, with limited focus on HRV responses to resistance training. The smartphone application that was used in this case study has been validated for recording the parasympathetic marker of RMSSD following a shortened time period of only 2 min: i.e., 1 min for stabilization and 1 min for recording [50]. Technology such as this has allowed the ability to determine the effects of training in competitive athletes and may be useful as part of a comprehensive monitoring protocol. Coaches and sport physiologists are encouraged to evaluate both the weekly RMSSD_MEAN_ and RMSSD_CV_ when interpreting HRV trends throughout training. Both markers may be important regarding adaptation. However, this case study showed the strong relationship between TV and RMSSD_CV_ in an individual strength trained athlete. This finding suggested that increases and decreases in RMSSD_CV_ may be found with respective increases and decreases in TV during a resistance training program. Therefore, HRV monitoring may be useful as a supplemental tool to help guide coaches when designing a periodized resistance training program for individual athletes. However, follow-up systematic investigation is needed to confirm and expand these findings before the widespread use of HRV monitoring for guiding strength training practices.

## Figures and Tables

**Table 1:** Estimation of 1-RM from 8-RM and 3-RM Testing (kgs).

Timeline	Squat	Bench	Deadlift
Pre	118.2	95.5	140.9
Mid	136.4	109.1	195.5
Post	145.5	109.1	197.7

**Table 2:** Exercises for each mesocycle throughout the 18-week resistance training program.

**Mesocycle 1 (W1–W5) & Mesocycle 2 (W6–W10)**		
Session 1	Session 2	Session 3
Back Squat	Bench Press	Deadlift
DB Bench Press	Leg Press	DB Incline Press
DB Rows	BB Low Rows	BB High Rows
Back Extensions	DB RDLs	Back Extensions
Forward Lunges	Lateral Lunges	Reverse Lunges
Sit-ups	Hanging Leg Raises	Russian Twists
**Mesocycle 3 (W11–W14) & Mesocycle 4 (W15–W18)**		
Session 1	Session 2	Session 3
Back Squat	Power Cleans	Push Press
BB RDLs	Bench Press	Deadlift
BB Incline Press	BB Rows	Pull-downs
Pull-ups	Back Extensions	Back Extensions
BB Forward Lunges	BB Lateral Lunges	BB Reverse Lunges
AB Flexion Machine	Hanging Leg Raises	Russian Twists

**Table 3:** Weekly measures of total volume-load, total load, total volume, and HRV.

Timeline	Total Volume-Load (AUs)	Total Load (kgs)	Total Volume (Reps)	HRV			
	Sum	Sum	Sum	Mean	% Δ	CV	% Δ
BL	-	-	-	77.79	-	16.40	-
Week 1	19,323.27	571.23	576	77.24	0.71	21.74	−32.59
Week 2	24,150.00	643.18	612	74.01	4.86	13.31	18.85
Week 3	25,689.09	670.09	618	72.51	6.79	18.65	−13.73
Week 4	30,302.73	648.50	667	70.41	9.49	12.58	23.29
Week 5	12,727.27	636.36	320	84.39	−8.48	7.31	55.44
Week 6	21,954.00	758.32	480	75.26	3.25	16.05	2.12
Week 7	25,980.91	805.27	510	81.07	−4.21	12.61	23.10
Week 8	23,759.45	691.68	540	86.56	−11.27	13.73	16.30
Week 9	36,815.91	887.50	641	80.67	−3.70	17.43	−6.27
Week 10	14,181.82	886.36	272	77.90	−0.14	10.50	35.97
Week 11	19,981.82	965.91	340	78.91	−1.44	9.44	42.44
Week 12	21,186.36	1002.27	350	-	-	-	-
Week 13	21,320.45	1052.27	351	74.61	4.09	5.87	64.19
Week 14	14,400.00	1054.55	218	70.05	9.96	6.15	62.48
Week 15	22,702.27	1088.64	359	75.87	2.47	4.68	71.43
Week 16	23,902.27	1138.64	361	69.95	10.08	8.66	47.21
Week 17	24,431.82	1177.27	363	68.38	12.09	11.82	27.95
Week 18	25,443.18	1206.82	368	75.66	2.74	11.05	32.64

Note: AUs = arbitrary units, kgs = kilograms, Reps = repetitions, HRV = heart rate variability, Mean represents RMSSD_MEAN_, CV = coefficient of variation, % Δ = percent change from baseline measure, BL = baseline, mesocycle 1 (W1–W5), mesocycle 2 (W6–W10), mesocycle 3 (W11–W14), and mesocycle 4 (W15–W18).

**Table 4:** Weekly mean ratings of perceived exertion (RPE) measures.

Time	RPE
Week 1	5.67
Week 2	6.67
Week 3	6.33
Week 4	8.00
Week 5	3.00
Week 6	5.00
Week 7	6.00
Week 8	7.33
Week 9	10.00
Week 10	4.00
Week 11	5.67
Week 12	6.67
Week 13	8.00
Week 14	4.00
Week 15	6.33
Week 16	7.67
Week 17	8.33
Week 18	9.00

## References

[1] Malik M. (1996). Heart rate variability. Ann. Noninvasive Electrocardiol..

[2] Wolf M.M., Varigos G.A., Hunt D., Sloman J.G. (1978). Sinus arrhythmia in acute myocardial infarction. Med. J. Aust..

[3] Aubert A.E., Seps B., Beckers F. (2003). Heart rate variability in athletes. Sports Med..

[4] Buchheit M. (2014). Monitoring training status with HR measures: Do all roads lead to Rome?. Front. Physiol..

[5] Flatt A.A., Esco M.R. (2013). Validity of the ithlete smart phone application for determining ultra-short-term heart rate variability. J. Hum. Kinet..

[6] Plews D.J., Laursen P.B., Stanley J., Kilding A.E., Buchheit M. (2013). Training adaptation and heart rate variability in elite endurance athletes: Opening the door to effective monitoring. Sports Med..

[7] Plews D.J., Scott B., Altini M., Wood M., Kilding A.E., Laursen P.B. (2017). Comparison of heart-rate-variability recording with smartphone photoplethysmography, polar H7 chest strap, and electrocardiography. Int. J. Sports Physiol. Perform..

[8] Esco M.R., Flatt A.A. (2014). Ultra-short-term heart rate variability indexes at rest and post-exercise in athletes: Evaluating the agreement with accepted recommendations. J. Sports Sci. Med..

[9] Flatt A.A., Esco M.R., Nakamura F.Y., Plews D.J. (2017). Interpreting daily heart rate variability changes in collegiate female soccer players. J. Sports Med. Phys. Fitness.

[10] Le Meur Y., Pichon A., Schaal K., Louis J., Gueneron J., Vidal P.P., Hausswirth C. (2013). Evidence of parasympathetic hyperactivity in functionally overreached athletes. Med. Sci. Sports Exerc..

[11] Plews D.J., Laursen P.B., Kilding A.E., Buchheit M. (2013). Evaluating training adaptation with heart-rate measures: A methodological comparison. Int. J. Sports Physiol. Perform..

[12] Buchheit M., Mendez-Villanueva A., Quod M.J., Poulos N., Bourdon P. (2010). Determinants of the variability of heart rate measures during a competitive period in young soccer players. Eur. J. Appl. Physiol..

[13] Figueiredo T., Willardson J.M., Miranda H., Bentes C.M., Reis V.M., Simão R. (2015). Influence of load intensity on postexercise hypotension and heart rate variability after a strength training session. J. Strength Cond. Res..

[14] Nakamura F.Y., Pereira L.A., Rabelo F.N., Flatt A.A., Esco M.R., Bertollo M., Loturco I. (2016). Monitoring weekly heart rate variability in futsal players during the preseason: The importance of maintaining high vagal activity. J. Sports Sci..

[15] Flatt A.A., Esco M.R. (2014). Endurance performance relates to resting heart rate and its variability: A case study of a collegiate male cross-country athlete. J. Am. Soc. Cytopathol..

[16] Plews D.J., Laursen P.B., Kilding A.E., Buchheit M. (2012). Heart rate variability in elite triathletes, is variation in variability the key to effective training? A case comparison. Eur. J. Appl. Physiol..

[17] Friedman K. (2016). Essentials of strength training and conditioning, 4th edition. Med. Sci. Sports Exerc..

[18] Lander J. (1984). Maximum based on reps. NSCA J..

[19] Richens B., Cleather D.J. (2014). The relationship between the number of repetitions performed at given intensities is different in endurance and strength trained athletes. Biol. Sport.

[20] Pereira M.I.R., Gomes P.S.C. (2003). Muscular strength and endurance tests: Reliability and prediction of one repetition maximum—Review and new evidence. Revista Brasileira de Medicina do Esporte.

[21] Hopkins L., Cochrane J., Mayhew J.L. (1993). Prediction of arm and leg strength from the 7-10-RM before and after strength training on Nautilus machine weights. IAHPERD J..

[22] Mayhew J.L., Ball T.E., Bowen J.C. (1992). Prediction of bench press lifting ability from submaximal repetitions before and after training. Sports Med. Train. Rehabil..

[23] Campos G.E., Luecke T.J., Wendeln H.K., Toma K., Hagerman F.C., Murray T.F., Ragg K.E., Ratamess N.A., Kraemer W.J., Staron R.S. (2002). Muscular adaptations in response to three different resistance-training regimens: Specificity of repetition maximum training zones. Eur. J. Appl. Physiol..

[24] Haff G. (2016). Essentials of Strength Training and Conditioning.

[25] Helms E.R., Cross M.R., Brown S.R., Storey A., Cronin J., Zourdos M.C. (2018). Rating of perceived exertion as a method of volume autoregulation within a periodized program. J. Strength Cond. Res..

[26] Cotter J.A., Garver M.J., Dinyer T.K., Fairman C.M., Focht B.C. (2017). Ratings of perceived exertion during acute resistance exercise performed at imposed and self-selected loads in recreationally trained women. J. Strength Cond. Res..

[27] Borg E., Kaijser L. (2006). A comparison between three rating scales for perceived exertion and two different work tests. Scand. J. Med. Sci. Sports.

[28] Flatt A.A., Hornikel B., Esco M.R. (2017). Heart rate variability and psychometric responses to overload and tapering in collegiate sprint-swimmers. J. Sci. Med. Sport.

[29] Heathers J.A. (2013). Smartphone-enabled pulse rate variability: An alternative methodology for the collection of heart rate variability in psychophysiological research. Int. J. Psychophysiol..

[30] Heathers J.A. (2014). Everything Hertz: Methodological issues in short-term frequency-domain HRV. Front Physiol..

[31] Flatt A.A., Esco M.R. (2016). Heart rate variability stabilization in athletes: Towards more convenient data acquisition. Clin. Physiol. Funct. Imaging.

[32] Carter J.R., Ray C.A., Downs E.M., Cooke W.H. (2003). Strength training reduces arterial blood pressure but not sympathetic neural activity in young normotensive subjects. J. Appl. Physiol..

[33] Gerhart H., Tai Y.L., Fennell C., Mayo X., Kingsley J.D. (2017). Autonomic modulation in older women: Using resistance exercise as a countermeasure. Int. J. Exerc. Sci..

[34] Kanegusuku H., Queiroz A.C., Silva V.J., de Mello M.T., Ugrinowitsch C., Forjaz C.L. (2015). High-intensity progressive resistance training increases strength with no change in cardiovascular function and autonomic neural regulation in older adults. J. Aging Phys. Act..

[35] Madden K.M., Levy W.C., Stratton J.K. (2006). Exercise training and heart rate variability in older adult female subjects. Clin. Investig. Med..

[36] Cooke W.H., Carter J.R. (2005). Strength training does not affect vagal-cardiac control or cardiovagal baroreflex sensitivity in young healthy subjects. Eur. J. Appl. Physiol..

[37] Esco M.R., Flatt A.A., Nakamura F.Y. (2016). Initial weekly HRV response is related to the prospective change in VO2max in female soccer players. Int. J. Sports Med..

[38] Flatt A.A., Esco M.R. (2015). Smartphone-derived heart-rate variability and training load in a women’s soccer team. Int. J. Sports Physiol. Perform..

[39] Smith D.L., Fernhall B. (2011). Advanced Cardiovascular Exercise Physiology.

[40] McArdle W.D., Katch F.I., Katch V.L. (2010). Exercise Physiology: Energy, Nutrition, and Human Performance.

[41] Lentini A.C., McKelvie R.S., McCartney N., Tomlinson C.W., MacDougall J.D. (1993). Left ventricular response in healthy young men during heavy-intensity weight-lifting exercise. J. Appl. Physiol..

[42] Falkel J.E., Fleck S.J., Murray T.F. (1992). Comparison of central hemodynamics between powerlifters and bodybuilders during resistance exercise. J. Strength Cond. Res..

[43] Fleck S.J., Dean L.S. (1987). Resistance-training experience and the pressor response during resistance exercise. J. Appl. Physiol..

[44] MacDougall J.D., Tuxen D., Sale D.G., Moroz J.R., Sutton J.R. (1985). Arterial blood pressure response to heavy resistance exercise. J. Appl. Physiol..

[45] Melo R.C., Quitério R.J., Takahashi A.C.M., Silva E., Martins L.E.B., Catai A.M. (2008). High eccentric strength training reduces heart rate variability in healthy older men. Br. J. Sports Med..

[46] Takahashi A.C., Melo R.C., Quitério R.J., Silva E., Catai A.M. (2009). The effect of eccentric strength training on heart rate and on its variability during isometric exercise in healthy older men. Eur. J. Appl. Physiol..

[47] Heffernan K.S., Fahs C.A., Shinsako K.K., Jae S.Y., Fernhall B. (2007). Heart rate recovery and heart rate complexity following resistance exercise training and detraining in young men. Am. J. Physiol. Heart Circ. Physiol..

[48] Kiviniemi A.M., Hautala A.J., Kinnunen H., Tulppo M.P. (2007). Endurance training guided individually by daily heart rate variability measurements. Eur. J. Appl. Physiol..

[49] Kiviniemi A.M., Hautala A.J., Kinnunen H., Nissilä J., Virtanen P., Karjalainen J., Tulppo M.P. (2010). Daily exercise prescription on the basis of HR variability among men and women. Med. Sci. Sports Exerc..

[50] Esco M.R., Flatt A.A., Nakamura F.Y. (2017). Agreement between a smartphone pulse sensor application and electrocardiography for determining lnRMSSD. J. Strength Cond. Res..

